# Health-related quality of life, cognitive screening, and functional status in a randomized phase III trial (EF-14) of tumor treating fields with temozolomide compared to temozolomide alone in newly diagnosed glioblastoma

**DOI:** 10.1007/s11060-017-2601-y

**Published:** 2017-08-28

**Authors:** Jay-Jiguang Zhu, Petya Demireva, Andrew A. Kanner, Susan Pannullo, Maximilian Mehdorn, Nicholas Avgeropoulos, Andrea Salmaggi, Antonio Silvani, Samuel Goldlust, Carlos David, Alexandra Benouaich-Amiel, Zvi Ram on behalf of the EF-14 Trial Investigators

**Affiliations:** 1 McGovern Medical School and Memorial Hermann Hospital at Texas Medical Center, University of Texas Health Science Center at Houston, Houston, TX USA; 2 TIRR Memorial Hermann, Houston, TX USA; 3 Rabin Medical Center, Sackler Faculty of Medicine, Tel Aviv University, Petah Tikva, Israel; 4 New York Presbyterian Hospital-Weill Cornell Medical Center, New York, NY USA; 5 Christian-Albrechts-University of Kiel, Kiel, Germany; 6 Univresity of Florida Health Cancer Center, Orlando, FL USA; 7 Istituto Neurologico C. Besta, Milano, Italy; 8 Foundation Neurological Institute “C. Besta”, Milan, Italy; 9 Hackensack University Medical Center, Hackensack, USA; 10 Lahey Clinic, Burlington, MA USA; 11 Hopitaux de Toulouse Purpan Pole Neurosciences PPR, Toulouse, France; 12 The Tel Aviv Sourasky Medical Center and Tel Aviv University Sackler School of Medicine, Tel Aviv, Israel

**Keywords:** Glioblastoma, Health-related quality of life (HRQoL), Cognition, Tumor treating fields, Temozolomide, EF-14

## Abstract

We characterized health-related quality of life (HRQoL), cognitive, and functional status in newly diagnosed glioblastoma (GBM) patients receiving Tumor treating fields (TTFields) with temozolomide (TMZ) versus TMZ alone in a planned interim analysis of a randomized phase III trial [NCT00916409], which showed significant improvement in progression-free and overall survival with TTFields/TMZ. After radiotherapy with concomitant TMZ, newly diagnosed GBM patients were randomized (2:1) to TTFields/TMZ (n = 210) or TMZ (n = 105). Interim analysis was performed in 315 patients with ≥18 months of follow-up. HRQoL, a secondary endpoint, was evaluated in per-protocol patient population and expressed as change from baseline (CFB) at 3, 6, and 9 months for each subscale in the EORTC QLQ-C30/BN20. Karnofsky performance scores (KPS) and Mini-Mental State Examination scores (MMSE) were assessed. CFB in HRQoL was balanced in treatment groups at the 12-month time point. Initially, HRQoL improved in patients treated with TTFields/TMZ (CFB3: 24% and CFB6: 13%) versus TMZ (CFB3: −7% and CFB6: −17%), though this difference was no longer evident at the 9-month point. General scales, including physical and social functioning, showed no difference at 9 and 12 months. TTFields/TMZ group reported higher concerns of “itchy skin”. KPS over 12 months was just below 90 in both groups. Cognitive status (MMSE) was stable over time. HRQoL, KPS, and MMSE were balanced in both groups over time. There was no preliminary evidence that HRQoL, cognitive, and functional status is adversely affected by the continuous use of TTFields.

## Introduction

Glioblastoma (GBM) is the most aggressive and common malignant primary brain tumor in adults, with an estimated 9600 to 11,200 new cases diagnosed in the US yearly [1]. Patients with GBM have a poor prognosis with median overall survival (OS) of 14.6 months in clinical trial settings and 11 months in general GBM population [2, 3], and patients experience recurrence on average within 7 months despite use of multimodal therapies [3, 4]. Given that novel treatment approaches for GBM have only offered modest gains in OS and/or progression-free survival (PFS), their role in improving functioning or palliating adverse symptoms has been highlighted, and thus assessment of neurocognitive functioning (NCF) and health-related quality of life (HRQoL) has gained priority in neuro-oncology trials [5].

Quality of life is a multi-faceted construct, encompassing physical, emotional, cognitive, and social functioning, and characterized by patient self-report to reflect a subjective assessment of well-being [6]. In addition to self-report, objective assessment of cognition through validated measures is also recommended, as it has been shown to relate to patients’ ability to perform basic and instrumental activities of daily living [7] and to have independent prognostic value for disease progression [8]. A large portion of patients with GBM experience reduced HRQoL, as well as decrement in objectively measured NCF in the pre-treatment setting [9]. Additional decline in HRQoL and NCF typically occurs throughout the course of the disease, secondary to side effects from therapies, tumor recurrence or progression, and disease related complications, such as seizures and venous thromboembolic events [6]. Therefore, measurement of HRQoL and NCF, as well as patient functional status via the Karnofsky Performance Score (KPS) at multiple time points have become standard in clinical trials for novel therapeutic approaches targeting glioma [5]. Furthermore, data regarding quality of life and cognition are factored into drug-efficacy metrics and can in turn inform physician and patient decision-making regarding choices of treatment [6].

Current standard-of-care treatment for newly diagnosed GBM includes maximum safe resection or biopsy and adjuvant concomitant radiation with temozolomide (TMZ) during the induction phase, followed by monthly TMZ for 6–12 months [3, 10]. Tumor Treating Fields (TTFields, Optune, Novocure, Ltd., Haifa, Israel) is a novel cancer therapy that leads to mitotic arrest and apoptosis of dividing cells by disrupting mitotic spindle formation during metaphase [11] and causing dielectrophoretic movement of polar molecules during cytokinesis [12]. The results from a randomized phase III trial (EF-11) comparing its efficacy with regard to extending OS and PFS in recurrent GBM in comparison to best physician chosen chemotherapy have been previously published [13]. Data from the trial indicated superior HRQoL for the TTFields group, pertaining to self-ratings of cognitive and emotional functioning in particular, as well as with regard to treatment related toxicity (appetite loss, constipation, diarrhea, nausea, and vomiting were more prevalent in the chemotherapy-treated arm). Most recently, a randomized phase III clinical trial (NCT00916409) for newly diagnosed GBM comparing TTFields/ TMZ versus TMZ alone has demonstrated superior outcomes of the treatment arm versus control arm in both OS and PFS at the preplanned interim analysis time point [14]. This report focuses on the findings detailing HRQoL, cognitive, and functional status in the same trial.

## Patients and methods

Full study details have been published previously [14]. Briefly, patients with newly diagnosed supratentorial GBM, age 18 and above, with KPS score of 70 or higher, with adequate bone marrow, liver, and renal functions were eligible for this trial. Patients were randomized at a ratio of 2:1 to receive TTFields/TMZ versus TMZ alone. Treatment with TTFields was initiated within 4–7 weeks from the last dose of the concomitant TMZ and radiation therapy. Randomization was performed through a central web-based randomization system and was stratified by extent of resection and O6-methyl guanine DNA methyltransferase (MGMT) gene methylation status. Raw scores on the HRQoL measures were normalized (range 0–100). A change of 10 points on a scale in either direction was regarded as the minimal clinically significant change [15].

Patients in the TTFields/TMZ group received continuous TTFields combined with cyclic TMZ per standard of care. TTFields was administered via placement of two pairs of transducer arrays on shaved scalp according to an individualized array map based on patient magnetic resonance imaging (MRI) and tumor location(s). The transducer array map was generated using mapping software designed for optimization of field intensity within the treated tumor (NovoTAL^®^, Novocure Ltd). The TTFields generate 200 kHz alternating electric fields within the brain. Following training in array placement, patients were supplied with transducer arrays in sterile packaging in order to replace arrays at home every 3 days. It was recommended that patients use the TTFields continuously to reach a goal of an average of 18 h per 24 h in a 4 week period; short treatment breaks were allowed. For those with tumor progression, second-line chemotherapy was offered per choice of the treating physician. In the TTFields/TMZ group, TTFields could be maintained until a second radiological progression or a maximum of 24 months. The study was approved by institutional review boards of all 83 participating institutions. All patients had reviewed and signed informed consent prior to enrollment in the study.

### Procedures

At baseline, in addition to undergoing contrast-enhanced MRI of the brain (within 2 weeks prior to the start of treatment), comprehensive assessments were performed (within 1 week of treatment initiation) including physical and neurological examinations, collection of laboratory parameters, and measurement of patient independence in activities of daily living with the KPS. Patients completed questionnaires assessing HRQoL and cognitive screening with the Mini-Mental Status Exam (MMSE) [16]. HRQoL was assessed with an EORTC quality-of-life questionnaire core-30 (QLQ-C30, Version 3), supplemented by the brain cancer module (BN20). Thereafter, MMSE and KPS assessments were repeated once monthly during office visits, and the HRQoL questionnaires were administered once every 3 months until progression or withdrawal from the trial.

The EORTC quality of life questionnaire C30 (EORTC QLQ-C30) is a general 30-item core measure used in many multi-national clinical trials with cancer patients [6] and is supplemented by the EORTC QLQ Brain Neoplasm 20 (BN20), which was developed specifically for patients with brain cancer. Robust reliability and validity have been established for both measures [17, 18]. Subscales within the EORTC QLQ-C30 include: (a) five functional scales—physical, role, emotional, cognitive, and social, as well as a global health status rating; (b) three symptoms scales—nausea, vomiting, and fatigue; and (c) six single-item scales—insomnia, appetite loss, constipation, diarrhea, dyspnea, and financial effect of tumor/treatment. The BN20 comprises four domain scores: visual disorder, motor dysfunction, communication deficit, and future uncertainty, and seven individual symptom items: seizures, difficulty with bladder control, weakness in legs, headache, drowsiness, hair loss, and itching. All measures were scored in accordance with the recommended standardized approach [19]. For the EORTC QLQ-C30, a higher positive score on a functional scale or on the global health status scale represents improved functioning, whereas higher scores on symptom scales and single item scales represent a high level of symptomatology/difficulty.

The MMSE is a brief cognitive screening measure that has been translated into multiple languages and is designed to sample orientation (place and time), registration, attention, recall, language, and visual construction with a maximum total score of 30 points [20]. It has been shown to have acceptable reliability and validity in patients with dementia, though lower reliability values have been recorded in relatively “healthy” community samples [20]. A cut-off of 27 points was chosen to discriminate between cognitively impaired versus cognitively intact participants in accordance with findings among individuals of Caucasian background with higher levels of education [21].

Prospective recording of adverse events was implemented in accordance with the National Cancer Institute’s Common Terminology Criteria (version 3.0) and began from initiation of treatment of either TTF/TMZ or TMZ alone until 2 months after discontinuation of treatment. The descriptive interim findings with regard to presence of adverse events in each study group have been reported previously [14].

Given that cognitive status, functional status, and health-related quality of life were secondary endpoints of the EF-14 trial, analysis was performed on the per protocol patient population: TTFields/TMZ (n = 196) and TMZ alone (n = 84). Descriptive data was expressed as change from baseline (CFB), which was calculated for each time point over the course of the first year post-randomization, and is presented in the “Results” section.

## Results

**Fig. 1:**
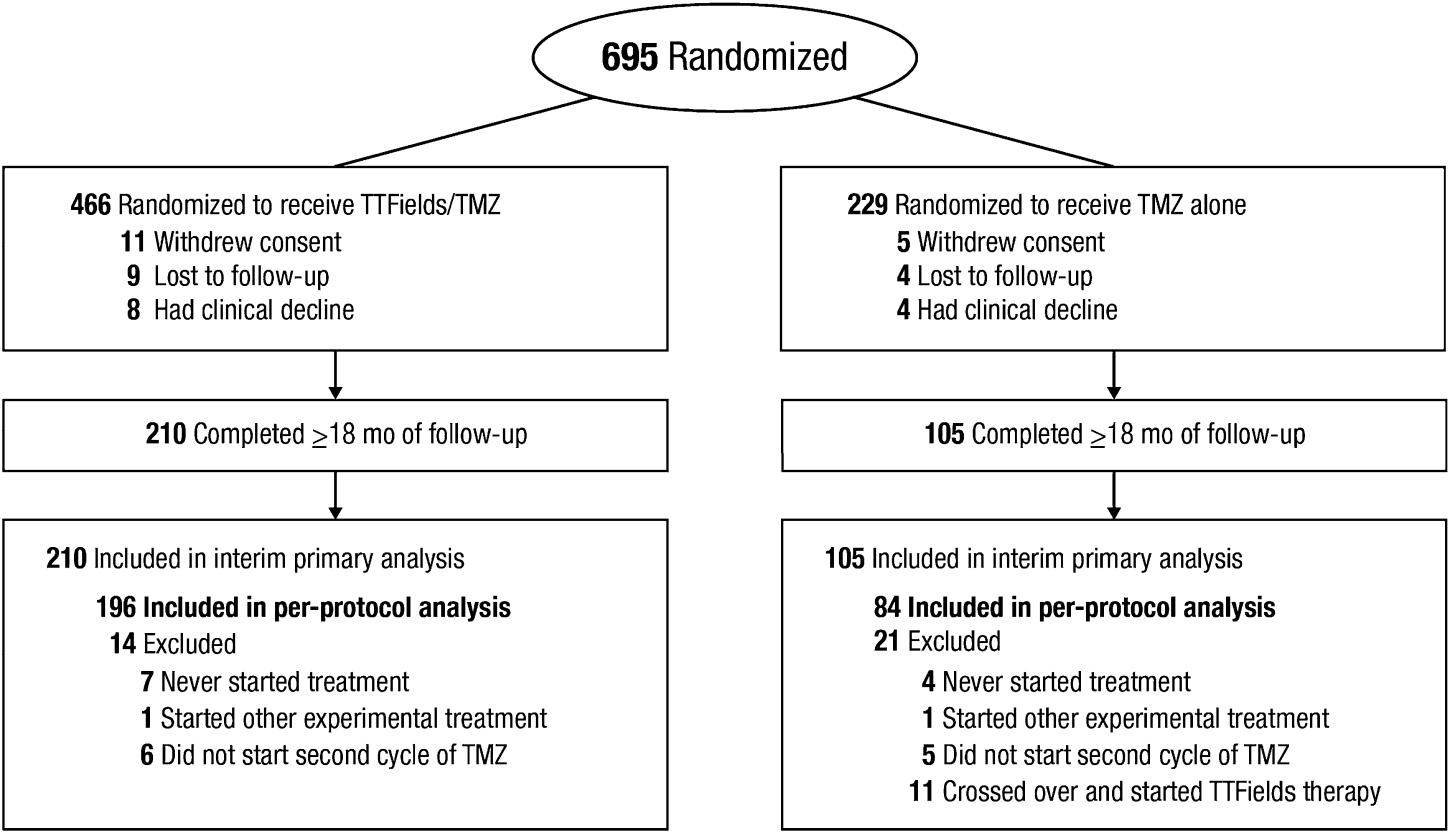
Patient flow

A total of 695 patients diagnosed with supratentorial GBM were randomized (2:1 ratio) to receive either TTFields/TMZ or TMZ alone between July 2009 and November 2014. As pre-specified, the interim analysis was performed when 210 and 105 patients were randomized to the TTFields/TMZ and TMZ alone, respectively, with a median follow-up of 38 months (range 18–60 months) (Fig. 1). The median time from randomization to treatment was 37 days (from the last day of radiotherapy to randomization). As the pre-defined boundaries for trial success (improvement of both PFS and OS) were met, the independent data and safety monitoring committee at the October 2014 meeting recommended termination of the study. The US Food and Drug Administration approved study termination; recruitment closed on November 29, 2014, when 695 of the planned 700 patients were enrolled. Patients in the TMZ alone arm were allowed to cross over to receive TTFields and follow-up of all patients continued in accordance with study protocol.

Patient baseline characteristics at interim analysis were well balanced between the two treatment groups (Table 1) [14]. Median age was 57 years and 66% were male. Tumor location was similar across groups. A gross total resection was achieved for 64% of the participants in each group, whereas about 11% had a biopsy in each arm. Use of antiepileptic medication and corticosteroid therapy was comparable across the two groups. Median KPS score at baseline was 90 for each group. Based on MMSE scores at baseline, 15 and 13% were classified as cognitively impaired at baseline in the TTFields/TMZ and TMZ groups, respectively.

**Table 1:** Baseline characteristics (per protocol population)

Baseline characteristics	TTFields/TMZ (n = 196) n (% or range)	TMZ (n = 84) n (% or range)
Age: median (range)	57 (20–83)	58 (21–80)
Sex: M/F	133/63 (67.9/32.1)	54/30 (64.3/35.7)
KPS: median (range)	90 (60–90)	90 (70–100)
Time from diagnosis to randomization (days)	116 (59–171)	113 (43–170)
Tumor location		
Both	2 (1.0)	1 (1.2)
Corpus Callosum	8 (4.1)	3 (3.6)
Left	87 (44.4)	32 (38.1)
Right	99 (50.5)	48 (57.1)
Extent of resection		
Biopsy	21 (10.7)	10 (11.9)
Gross total resection	127 (64.8)	54 (64.3)
Partial resection	48 (24.5)	20 (23.8)
Cycles of TMZ (Median and range)	6 (1–26)	4 (1–24)
Cycles of TTFields	9 (1–58)	0 (0)

HRQoL assessment was a secondary endpoint of the study and was analyzed in the per protocol population. Compliance with questionnaire (EORTC QLQ-C30 and BN20) completion was 82% (173/210) in the TTFields/TMZ group and 64% (67/105) in the TMZ alone group. All assessments were carried out until the number of remaining patients was ≤70.

### Functional and cognitive status

**Fig. 2:**
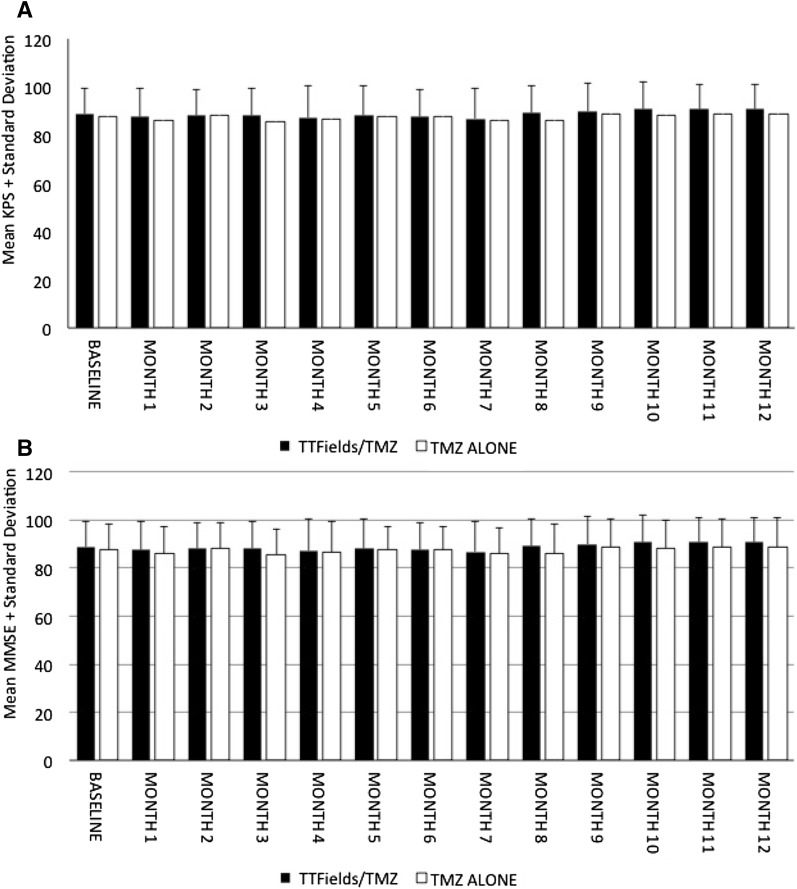
Mean changes in KPS (**a**) and MMSE (**b**) over 12 months with TTFields/TMZ versus TMZ alone. (Per Protocol Population)

KPS ratings at baseline and monthly thereafter reflected relative stability with regard to patient independence in activities of daily living over time during the first year after randomization in either group; the majority of mean scores remained just under 90 (Fig. 2a). Mean percentage change from baseline (CFB) was between −4.3 (month 7) and −1.6 (month 1) within the TTFields/TMZ group and between −4.2 (month 8) and −0.4 (month 2) in the TMZ alone group. Similarly, mean cognitive status within either group did not decline below 27/30 and no differences in MMSE scores were documented between the groups (Fig. 2b). Mean percentage change in MMSE scores ranged from −2.4 (month 1) to 4.8 (month 8) in the TTFields/TMZ arm, and from −0.5 (month 2) to 3.8 (month 8) in the TMZ alone group.

### Health-related quality of life (HRQoL)

**Fig. 3:**
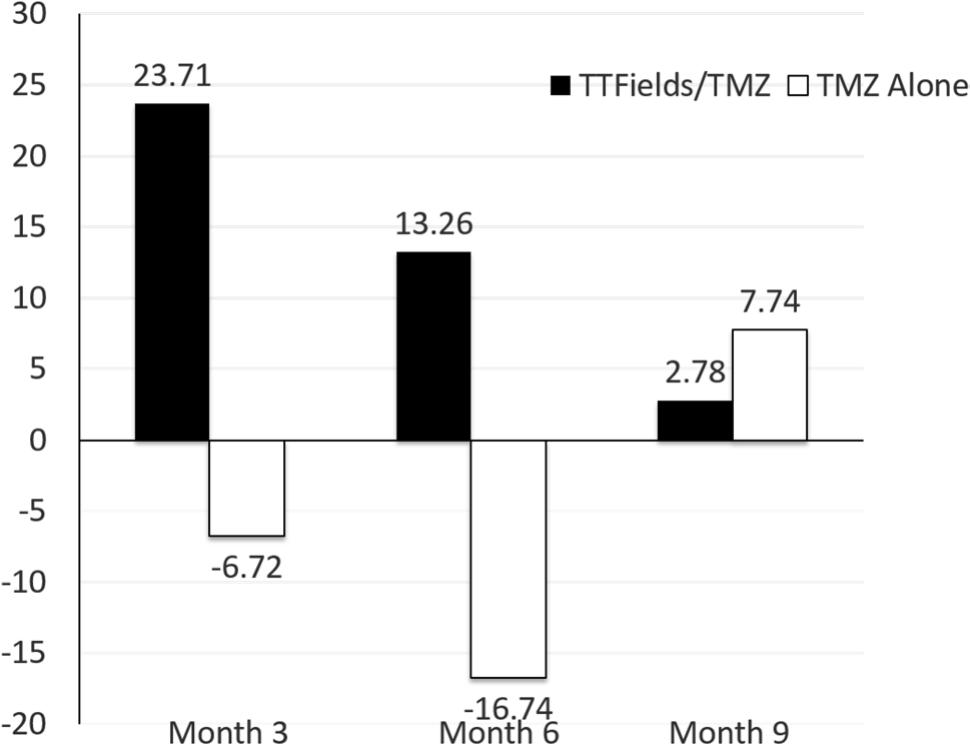
Changes from baseline (%) in Global Health Status from baseline to 9 months with TTFields/TMZ versus TMZ alone. (Per Protocol Population). An increase in percentage corresponds to an increase in QoL

**Fig. 4:**
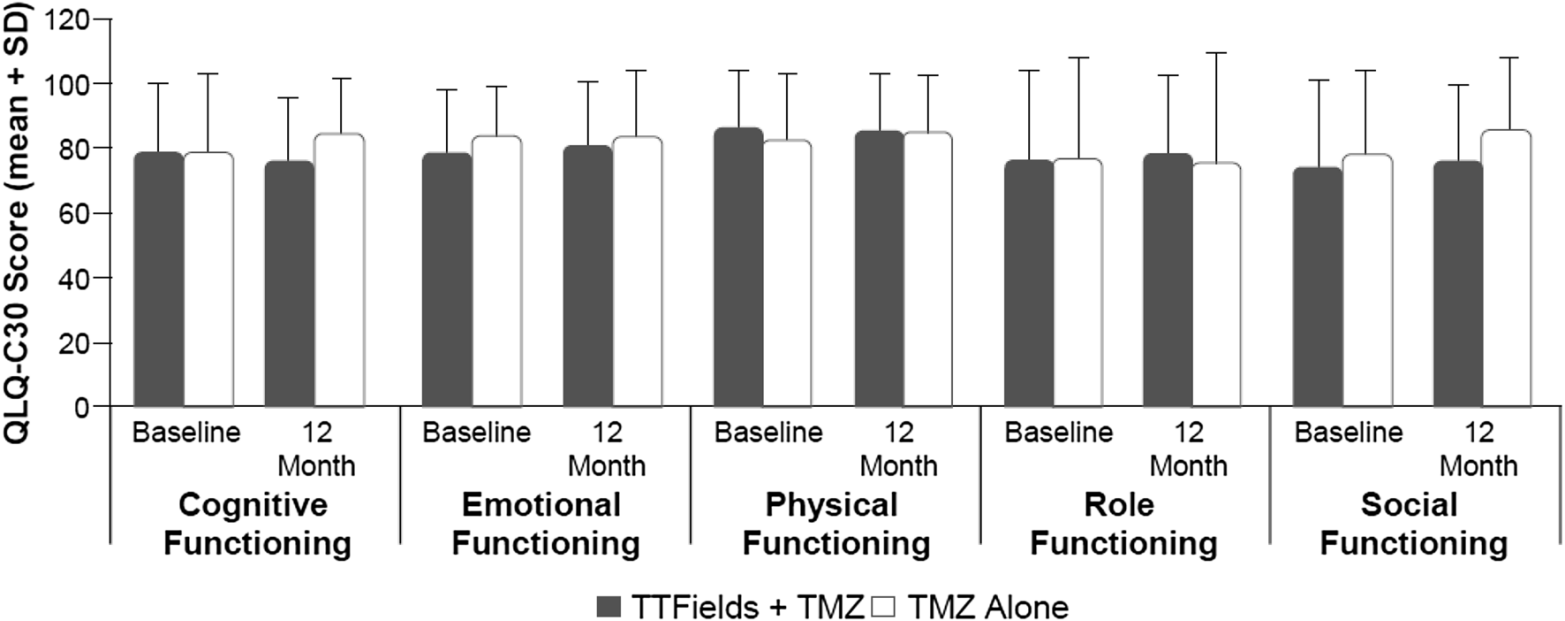
QLQ-C30 Global Health Status and General Scales in TTFields/TMZ versus TMZ alone groups over 12 months in Per Protocol Population. EORTC QLQ-C30, European Organisation for Research and Treatment of Cancer Quality of Life Questionnaire-C30; *SD* standard deviation, *TMZ* temozolomide, *QoL* quality of life

**Fig. 5:**
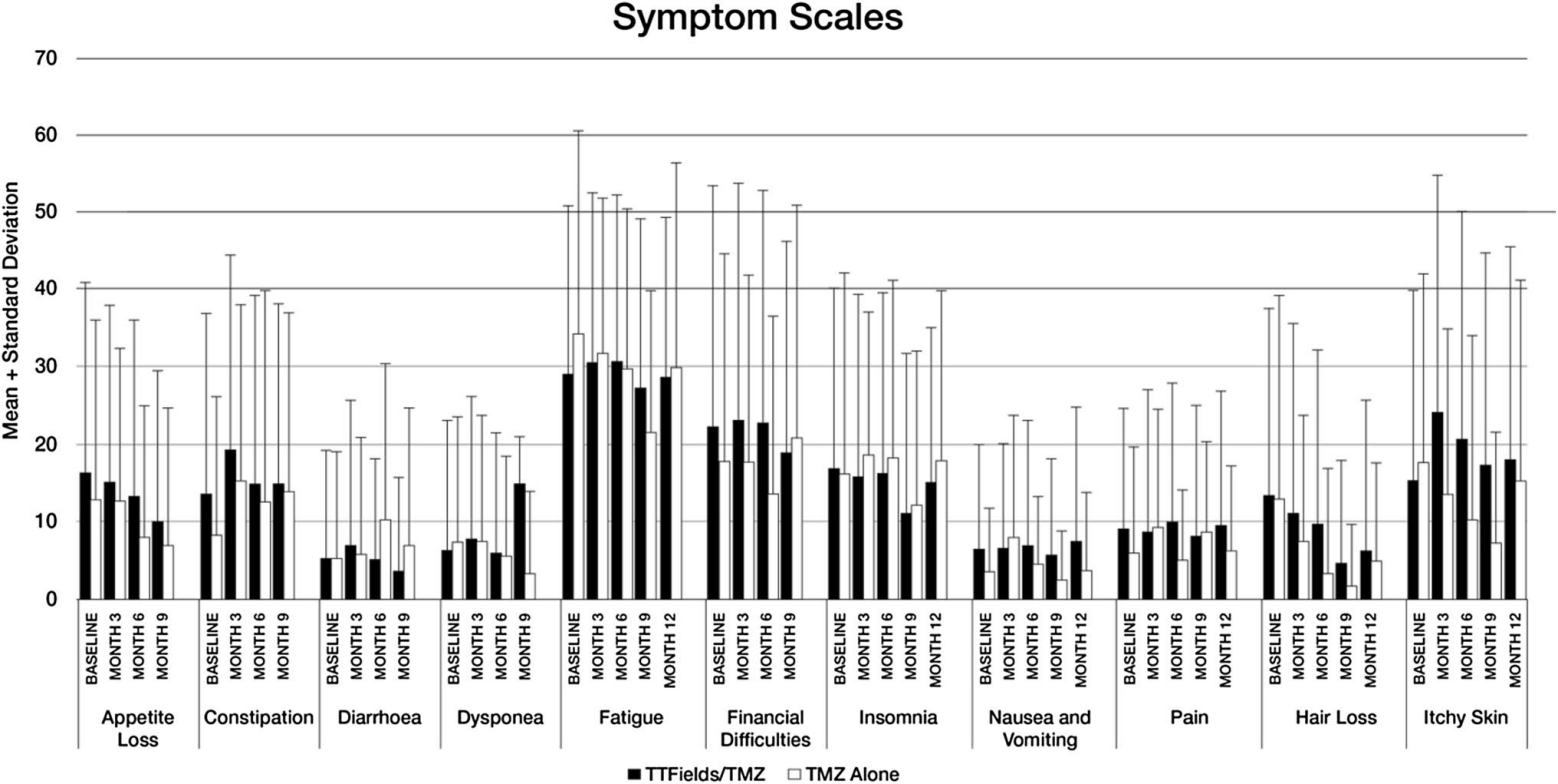
QLQ-C30 Symptom Scales in TTFields/TMZ versus TMZ alone groups over 12 months in Per Protocol Population. An increase in percentage corresponds to a decrease in QoL

At 3 and 6 months, HRQoL initially improved in patients treated with TTFields/TMZ (CFB3: 24% and CFB6: 13%) versus mild reduction in the TMZ group (CFB3: −7% and CFB6: −17%). However, at 9 months the CFB were 0.42 (2.9%) in the TTFields/TZM group, and 0.0 (7.8%) in the TMZ group **(**Fig. 3 **)**. There were no significant changes registered from baseline values on any of the quality-of-life scales (Fig. 4). No significant group differences were reported for any of the functional scales from the EORTC QLQ-C30 measure. Patients did not rate their physical or social functioning any differently when receiving TTFields/TMZ versus TMZ alone. Group differences were pronounced for “itchy skin,” where patients receiving TTFields/TMZ had higher numeric scores (Fig. 5). Self-reported neurologic symptomatology on BN20 did not differ between the two groups and as expected reflected a tendency toward increase in symptoms, likely related to known side effects of TMZ, which was administered to both groups.

## Discussion

The current study reviewed available data at the interim analysis from the first multicenter international phase III clinical trial of TTFields added to standard-of-care therapies in patients with newly diagnosed glioblastoma (EF-14). Although findings characterizing the efficacy of TTFields with regard to OS and PFS have been encouraging and were published elsewhere [14], this is the first report of cognitive status, self-reported HRQoL and performance status at the pre-planned interim analysis of the trial. As such, the data presented herein may offer important implications for the provision of clinical care to individuals with newly diagnosed glioblastoma and their families and highlights directions for future study.

Interim review of the data revealed no significant group differences across any of the administered measures, including mean cognitive status, KPS-assessed functional status, and HRQoL. Results indicated that patients receiving TTFields/TMZ and TMZ alone, on average, had intact cognitive status (all means stayed above the 27-point cut-off for both groups) and were independent with regard to functional status (KPS mean scores at 3, 6, 9, and 12 months following randomization remained just below 90). Mean self-reported functional scales from the EORTC QLQ-C30 did not differ between the two groups, including self-ratings of social functioning and physical functioning, which could theoretically be adversely impacted by wearing the TTFields device. Findings from the cognitive screening (MMSE) showing no adverse change in cognitive status were congruent with results from HRQoL measures revealing no significant CFB in self-ratings of cognitive functioning. Similarly, self-reported emotional functioning was also not significantly different relative to baseline in both groups at months 3, 6, and 9. Although symptoms known to be associated with TMZ treatment (e.g., nausea/vomiting, diarrhea, constipation, appetite loss, fatigue and pain) were reported by both groups, no differences were noted between treatment groups.

The present HRQoL findings are consistent with results from the previous phase III trial (EF-11) assessing efficacy and HRQoL in patients with recurrent GBM treated with TTFields versus physician best choice chemotherapy [13]. Similarly, there were no differences between the TTFields group and patients receiving chemotherapy on self-ratings of global health and social functioning; ratings of cognitive and emotional functioning were higher in the TTFields group, and role functioning favored TTFields. In addition, pain and fatigue were increased only in the chemotherapy patients, but not in the patients receiving TTFields.

Limitations inherent in the design of the current clinical trial have been addressed elsewhere and comprise: exclusion of patients with very poor prognoses due to the timing of randomization; reporting bias given that patients in the TTFields group were allowed to remain in the trial beyond progression; and absence of a sham/placebo treatment [14]. Compliance with questionnaire (EORTC QLQ-C30 and BN20) completion was 82% (173/210) in the TTFields/TMZ group and 64% (67/105) in the TMZ alone group due to more patients in the TMZ arm withdrawing consent after GBM progression to participate in other trials, whereas patients in the TTFields/TMZ arm continued treatment and clinical follow up with MMSE and KPS collected monthly during office visits, and HRQoL questionnaires completed every 3 months until a second progression. It can be extrapolated that missing data from noncompliant participants in the TMZ group would likely skew toward poorer ratings for HRQoL and reduced cognitive status, given findings of declining functioning with advancement of disease. Nevertheless, increased efforts to collect HRQoL data, and perform cognitive screening and assessment of functional status as participants exit the trial, in addition to the measurements collected at assigned study time intervals, are recommended in order to provide more complete comparisons between treatment modalities.

The use of the MMSE to measure cognition in clinical trials of patients with primary brain tumor has been questioned on grounds of poor sensitivity, potential practice effects with repeated administration, and the failure to adequately sample frontal subcortical functions impacted by radiotherapy [22]. Brevity, low cost, and ease of administration during a typical office visit has contributed to wide utilization of MMSE in multiple phase II and phase III clinical trials. It is noted that, had the current study utilized neurocognitive measures with higher sensitivity, as has been suggested in other work [23, 24], greater decline from baseline in cognition may have been documented in both treatment groups, consistent with levels registered in other investigations. The MMSE has been demonstrated to capture cognitive status deterioration in patients receiving escalating doses of radiation [23], hypofractionated radiation with TMZ [24], as well as in patients experiencing first progression of GBM while enrolled in a bevacizumab plus standard-of-care clinical trial [25]. MMSE scores have also been shown to correlate to scales assessing activities of daily living [25]. Nevertheless, given the demonstrated feasibility of more sensitive neurocognitive measures and explicit recommendations by the International Cognition and Cancer Task Force [26], further investigation of potential neurocognitive impact of TTFields on patients with GBM is warranted in future studies. In addition, in light of the high KPS and MMSE scores recorded in both treatment groups in the present study, future investigations in patients with GBM where cognitive deficits may range from very subtle to severe are cautioned to exercise care in employing neurocognitive tests with demonstrated sensitivity to milder decline in cognitive and performance status.

In summary, prospective measurement of HRQoL and neurocognitive functioning as part of clinical trials evaluating novel treatments for GBM has become standard in recent years. It is critical to adequately monitor treatment-related cytotoxicity and provide a multifaceted assessment of the impact of the illness and treatments on patient daily functioning, particularly in light of the limited survival duration for these individuals. As further advances are made in the GBM treatment arena, such data would be essential in providing guidelines regarding expected side effects and informing physician and patient choices of preferred treatment modality.
